# The Critical Raw Materials Issue between Scarcity, Supply Risk, and Unique Properties

**DOI:** 10.3390/ma14081826

**Published:** 2021-04-07

**Authors:** Mihaela Girtan, Antje Wittenberg, Maria Luisa Grilli, Daniel P. S. de Oliveira, Chiara Giosuè, Maria Letizia Ruello

**Affiliations:** 1 Photonics Laboratory, (LPhiA) E.A. 4464, SFR Matrix, Faculté des Sciences, Université d’Angers, 2 Bd Lavoisier, 49000 Angers, France; 2 Bundesanstalt für Geowissenschaften und Rohstoffe (BGR), Stilleweg 2, 30655 Hannover, Germany; antje.wittenberg@bgr.de; 3 Energy Technologies and Renewable Sources Department, ENEA-Italian National Agency for New Technologies, Energy and Sustainable Economic Development, Casaccia Research Center, Via Anguillarese 301, 00123 Rome, Italy; marialuisa.grilli@enea.it; 4 Laboratório Nacional de Energia e Geologia, Mineral Resources and Geophysics Research Unit, Estrada da Portela, Bairro do Zambujal, Apartado 7586, Alfragide, 2610-999 Amadora, Portugal; daniel.oliveira@lneg.pt; 5 Mineral Resources Expert Group, EuroGeoSurveys, Rue Joseph II, 36-38, Box 7, 1000 Brussels, Belgium; 6 Department of Materials, Environmental Sciences and Urban Planning (SIMAU), Università Politecnica delle Marche—INSTM Research Unit, 60131 Ancona, Italy; c.giosue@staff.univpm.it

**Keywords:** Critical Raw Materials (CRM), substitution, extreme conditions, mining, scarce minerals, supply chains, strategic elements, universe-abundant elements

## Abstract

This editorial reports on a thorough analysis of the abundance and scarcity distribution of chemical elements and the minerals they form in the Earth, Sun, and Universe in connection with their number of neutrons and binding energy per nucleon. On one hand, understanding the elements’ formation and their specific properties related to their electronic and nucleonic structure may lead to understanding whether future solutions to replace certain elements or materials for specific technical applications are realistic. On the other hand, finding solutions to the critical availability of some of these elements is an urgent need. Even the analysis of the availability of scarce minerals from European Union sources leads to the suggestion that a wide-ranging approach is essential. These two fundamental assumptions represent also the logical approach that led the European Commission to ask for a multi-disciplinary effort from the scientific community to tackle the challenge of Critical Raw Materials. This editorial is also the story of one of the first fulcrum around which a wide network of material scientists gathered thanks to the support of the funding organization for research and innovation networks, COST (European Cooperation in Science and Technology).

## 1. Introduction

The European Union (EU) has a long history of extracting, processing, and producing raw materials. Modern and optimized mining technologies and resource-efficient production are a reality at many European mining sites, and yet, the import dependency for many raw materials sourced outside the EU has dramatically increased over the last decades. With the Raw Materials Initiative, which was started in 2008 and consolidated in 2011 [1,2], the EU restarted actions to secure the global competitiveness of the manufacturing industries and to accelerate the transition to a resource-efficient and sustainable society. The Raw Materials Initiative has three pillars with the following aims: (i) ensure fair and sustainable supply of raw materials from global markets; (ii) foster sustainable supply of raw materials within the EU; and (iii) boost resource efficiency and supply of “secondary raw materials” through recycling.

**Figure 1:**
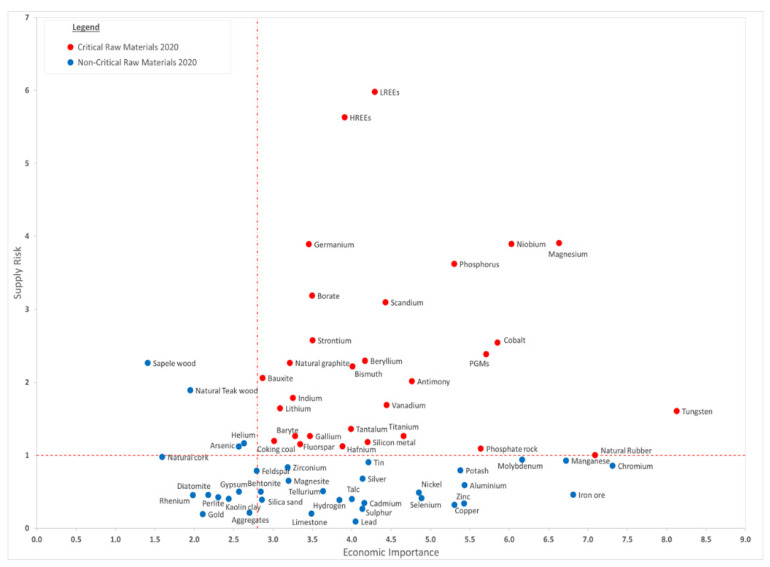
Critical Raw Materials (CRMs) chart based on the 2020 CRM list. Reprinted from [3] Copyright from European Commission. LREEs and HREEs stand for light and heavy rare earth elements (REEs), respectively.

Critical Raw Materials (CRMs) are one of key subjects for the EU economy. The European Commission published the first list of CRMs for the EU in 2011 [2]. Yet, the concept of criticality is very dynamic and, hence, the list of these CRMs undergoes a triennial revision. The latest list (2020) [3,4] representing the economic importance and associated supply risk is given in Figure 1 and shows that 30 elements or groups of elements are in the critical area as compared to the 14 materials in 2011, 20 materials in 2014 [5], and 27 materials in 2017 [6].

The CRMs list combines raw materials of high importance to the EU economy and their associated supply risk. Most of the CRMs have unique characteristics that make them very difficult to be substituted in the selected application; therefore, their substitutability remains still an issue [3,7].

Securing access to metals and minerals that are strategic for high-tech products is a global concern, and it is common also to other highly industrialized countries, such as Japan and the United States, whose high-tech products are strongly dependent on the import of raw materials. In 2011, the EU started also a trilateral initiative with Japan and US to promote cooperation in critical raw material issues. Despite the different domestic approaches to CRMs from the above-mentioned countries, since 2011, the EU, US, and Japan representatives meet every year in a one-day conference for discussing and updating about raw materials data, analysis of trade, and recycling and substitution issues.

According to analyses that recognize increasing pressure on resources, roadmaps of several initiatives, networks, and EU-funded projects were established.

Among the initiatives, we mention the European Innovation Partnership (EIP) on Raw Materials, which is a platform that brings together representatives from industry, public services, academia, and non-governmental organizations (NGOs) with the aim of reinforcing the Raw Materials Initiative by mobilizing the stakeholder community to implement actions for reducing Europe’s import dependency on critical raw materials strategic for the industrial sector, while focusing also on the societal benefit of research and innovation (R&I) activities. Its mission is to provide high-level guidance to the European Commission, EU countries, and private actors on innovative approaches to the challenges related to raw materials. To address the EIP’s initiative, several EIP Commitments were established. Commitments are networks undertaken by several partners who commit themselves to activities aimed at achieving the EIP’s objectives. The first call for EIP Commitment was in 2013 and in 2017, already 127 EIP Commitments were settled up [8]. The last call was in 2019.

The EIT (European Institute of Innovation and Technology) Raw Materials is the largest consortium in the raw materials sector worldwide, comprising more than 120 members. Its mission is to enable sustainable competitiveness of the European minerals, metals, and materials sector along the value chain by driving innovation, education, and entrepreneurship. It was initiated and funded by the EIT (European Institute of Innovation and Technology), which is a body of the European Union. The EIT boosts education and encourages new entrepreneurs and innovators to turn their innovative ideas into business opportunities, funding several projects on the whole raw materials value chain. The EIT is the largest consortium in the raw materials sector worldwide [9].

The European Battery Alliance (EBA) was launched in 2017 by the European Commission, EU countries, industry, and the scientific community. The European Battery Alliance aims to develop an innovative, competitive, and sustainable battery value chain in Europe. Securing access to raw materials for batteries is one of the six priority areas [10].

The European Raw Materials Alliance (ERMA)—launched 29 September 2020—aims to build resilience and strategic autonomy for Europe’s rare earth and magnet value chains. The support of Europe’s raw materials industry capability to extract, design, manufacture, and recycle materials is part of the actions. The ERMA is part of an Action Plan on Critical Raw Materials and the publication of the 2020 List of Critical Raw Materials [11].

We list below some of the EU and EIT-funded projects and EIP on CRM-related issues, which focus on technical solutions or on information on deposits, supply, and demand. The list is obviously non-exhaustive and reports only about some of the projects in which the authors of this manuscript were involved and/or linked:

- EURARE (Development of a sustainable exploitation scheme for Europe’s Rare Earth ore deposits), 1 January 2013–31 December 2017, aiming to set the basis for the development of a European rare earth elements REE industry that will safeguard the uninterrupted supply of REE raw materials and products crucial for the EU economy industrial sectors. Grant agreement ID: 309,373 co-funded by the European Commission (EC) under the 2012 Cooperation Work Programme for Nanotechnologies, Materials, and new Production Technologies and specifically the raw materials topic NMP.2012.4.1-1 “New environmentally friendly approaches in minerals processing” [12].
- FRAME (Forecasting And Assessing Europe’s Strategic Raw Materials Needs) and MINDeSEA (Seabed Mineral Deposits in European Seas: Metallogeny and Geological Potential for Strategic and Critical Raw Materials) these two scientific projects of GeoERA (Establishing the European Geological Surveys Research Area to deliver a Geological Service for Europe), 1 July 2018–31 December 2021, aiming to expand the strategic and CRM knowledge through a compilation of mineral potential and metallogenic areas of critical raw materials resources in Europe, which is focused on related metal associations on land and the marine environment. The GeoERA project has received funding from the European Union’s Horizon 2020 research and innovation program under grant agreement No. 731,166 [13].
- SUBST-EXTREME (Sustainable substitution in extreme conditions) is an EIP Commitment, 1 January 2014–30 December 2019, which develops substitutes for CRMs in energy, aerospace, and mining industries. Materials used in these industries at this moment are heat-resistant alloys, stainless steels, and hard materials. The whole value chain is present in this Raw Material Commitment, RMC, covering research/development, manufacturing, and end-users [14,15].
- ProSUM (Prospecting Secondary raw materials in the Urban mine and Mining wastes), 1 January 20153–1 December 2017, provides a factual basis for policy makers to design appropriate legislation, academia to define research priorities and to identify innovation opportunities in recovering CRMs for the recycling industry. The ProSUM project has received funding from the European Union’s Horizon 2020 research and innovation program under grant agreement N° 641,999 [16].
- SMART GROUND (Smart Ground–SMART data collection and inteGRation platform to enhance availability and accessibility of data and infOrmation in the EU territory on SecoNDary Raw Materials) project, 1 October 2015–31 March 2018, intended to address the issue of waste management and resource recovery from industrial, mining, and municipal landfills, improving the availability and accessibility of data and information on Secondary Raw Materials (SRM) in the EU, while creating synergies among the different stakeholders involved in the SRM value chain. The SMART GROUND project has received funding from the European Union’s Horizon 2020 research and innovation program under Grant Agreement No. 641,988 [17,18].
- MSP-REFRAM (Multi-Stakeholder Platform for a Secure Supply of Refractory Metals in Europe), 1 December 2015–31 August 2017, the project goal is to create a platform composed of multiple stakeholders to a secure supply of refractory metals in Europe, program H2020 Enabling the transition towards a green economy and society through eco-innovation. Grant agreement ID: 688,993 [19].
- VERAM (Vision and Roadmap for European Raw Materials), 1 December 2015–31 May 2018, aimed to produce a common medium-term (2030) and long-term 2050 vision and roadmap for relevant raw materials including metals, industrial minerals, and aggregates and wood that will enhance international cooperation on raw material policies and investments. The VERAM project has received funding from the European Union’s Horizon 2020 research and innovation program under grant agreement No 690,388 [20,21].
- SUPERMAT (The VIRTUAL Centre for Integration of INNOVATIVE synthesis and Processing methods for SUSTAINABLE advanced Materials operating under Extreme Conditions), 1 January 2016–31 December 2018, its expected results include elaboration of innovative technologies based on thermodynamic prediction methods enabling sustainable synthesis of novel systems and validation in pilot scale conditions, reducing or even fully replacing CRMs in extreme environments applications. Grant agreement ID: 692,216 [22].
- MIN-GUIDE (Minerals Policy Guidance for Europe), 1 February 2016–31 January 2019, is a project addressing the need for a secure and sustainable supply of minerals in Europe by developing a ‘Minerals Policy Guide’. Grant agreement ID: 689,527 [23].
- Flintstone2020 “Next generation of superhard non-CRM materials and solutions in tooling”, 1 February 2016–31 January 2020, aims to provide a perspective for the replacement of two important CRMs—tungsten (W) and cobalt (Co)—which are the main constituents for two important classes of hard materials (cemented carbides/WC-Co, and PCD/diamond-Co), by developing innovative alternative solutions for tooling operating under extreme conditions. The Flintstone2020 project has received funding from the European Union’s Horizon 2020 research and innovation program under grant agreement No. 689,279 [24].
- EQUINOX (A novel process for manufacturing complex shaped Fe-Al intermetallic parts resistant to extreme environments), 1 February 2016–31 July 2019, has as its main objective developing a novel near net shape production technology that allows substituting stainless steel parts in high volume end consumer markets by a new class of CRM-free, ductile Fe-Al based intermetallics. Grant agreement ID: 689,510 [25].
- CRM-EXTREME (Solutions for Critical Raw Materials Under Extreme Conditions), May 2016 to April 2020, is a COST action-focused project on the possibility of the substitution of CRMs (such as Cr, Co, Nb, W, Y, and other rare earth elements) in high value alloys and metal–matrix composites used under extreme conditions of temperature, loading, friction, wear, and corrosion, in Energy, Transportation, and Machinery manufacturing industries. This action originated another project: ITHACA (Innovative and sustainable TecHnologies for reducing critical raw mAterials dependence for Cleaner transportation Applications), a COST INNOVATORS’ GRANT (CIG), which is one of the four CIGs funded for the first time by the COST Association in 2019 [26,27].
- MINEA (Mining the European Anthroposphere), May 2016–April 2020, a COST Action that aims to actuate the reporting of material resources/reserves in the anthroposphere. The focus is on (1) construction and demolition waste, (2) waste regained from landfills, and (3) solid residues from waste incineration [28].
- FORAM (Towards a World Forum on Raw Materials), 1st November 2016–31st October 2018, funded by the European Union’s Horizon 2020 research and innovation programme, helps developing a platform of international experts and stakeholders. FORAM aims to share experiences and increase understanding of all aspects of trade in raw materials [29].
- SCALE (Production of Scandium compounds and Scandium Aluminum alloys from European metallurgical by-products), 1 December 2016–31 May 2021, aims toward the efficient exploitation of EU high concentration scandium containing resources to develop a stable and secure EU scandium supply chain to serve the needs of EU aerospace and high tech industry. Grant agreement ID: 730,127 [30].
- SCRREEN (Solutions for CRitical Raw materials-a European Expert Network), 1 November 2016–31 December 2019, established six Expert Groups within an EU Expert Network that provided expertise on market, governments and policies, resources, circular economy, production, substitution, CSA coordination, and support action. Grant agreement ID: 730227. There is also the ongoing project SCRREEN 2 (Solutions for CRitical Raw materials-a European Expert Network 2), 1 November 2020–31 October 2023, aiming to continue to provide expertise [31].
- Minerals4EU (EU Minerals Intelligence Network for Europe), 1 September 2013–31 August 2015, developed a structure that delivered a web portal, a European Minerals Yearbook, and foresight studies followed by the project MICA (The Mineral Intelligence Capacity Analysis), 1 December 2015–31 January 2018, which contributed to raw materials knowledge infrastructure at the EU level and was followed by EUMINET (European Mineral Information Network), 1 May 2014–01 January 2020. With EGDI, the European Geological Survey Organizations provide a data infrastructure that hosts that information and its updates by GeoERA scientific projects. FP7-NMP-2013-CSA-7. Grant agreement ID: 608,921 [32].
- BlueMining (Sustainable extraction of raw materials from oceanic depths), 1 February 2014–31 January 2018, addressed challenges associated with the extraction of deep sea minerals, ranging from their discovery and assessment to exploitation technologies and the necessary legal and regulatory framework. Grant agreement ID: 604,500 [33].
- MinFuture (Global material flows and demand-supply forecasting for mineral strategies), 1 December 2016–30 November 2018, aims to identify, integrate, and develop expertise for global material flow analysis and scenario modeling. MinFuture is funded by the Horizon 2020 Framework Programme of the European Union under Grant Agreement no. 730,330 [34].
- EXTREME (Substitution of CRMs in components and coatings used under extreme conditions), a project founded by the KIC EIT Raw Materials, is a network of European infrastructures owned by partners with skills and expertise on the substitution/reduction of CRMs used under challenging conditions of temperature, wear, friction, loading, corrosion, etc., that are easily reached in several technological and industrial fields, such as manufacturing, machining, transport, and construction sectors [35].
- MONAMIX (New concepts for efficient extraction of mixed rare earths oxides from monazite concentrates and their potential use as dopant in high-temperature coatings and sintered materials) project addresses the topic 2 of ERAMIN II call: Design: 2.1: Product design for increased raw material efficiency and 2.4: Product design for critical materials substitution. This project deals with a hydro-chemical method for monazite concentrates purification by selective leaching and their use for hydrothermal synthesis of mixed nanostructured zirconia doped with different REOs/ZrO_2_ [36].

The indisputable conclusion after about 10 years of finalized CRM projects research is that the most advanced technologies required for the green and digital transition will lead to a drastic increase demand for certain CRMs in Europe and elsewhere [37].

Each updated CRMs report at the moment of its publication is ironically outdated, since it is based on a picture covering the previous last five years of a varying geopolitical scenario, and that the SARS-CoV-2 pandemic has shown that countries are more interrelated than the concept of globalization had made us aware.

Even if the action plan on CRMs looks at the current and future challenges and proposes viable actions to reduce Europe’s dependency on third countries, it would be interesting to examine more defined scenarios: (i) the natural abundance and scarcity of elements on Earth and the Universe and (ii) the availability from EU sources.

## 2. Abundance and Scarcity of Elements on Earth and Universe

Among the elements represented in Figure 1, which are presented as elements with important supply risk in the EU and of major importance for the economy, we have selected (in order of their atomic number Z) the following: Li, Be, B, P, Sc, Ti, V, Cr, Co, Ga, Ge, Se, Sr, Nb, In, Sb, Te, Ta, W, Bi, and rare earth elements. The main applications and main extraction and production countries of these elements are given in Table 1.

Understanding the scarcity of certain materials necessarily involves the comprehension of the mechanisms of the formation of elements and their abundance on the scale of the Earth, the Sun, or the Universe. Hence, if we accept the nucleosynthesis hypothesis and taking into account that the most abundant element in the Universe is hydrogen, one can imagine that by consecutive fusion, nuclear reactions formed all the known elements up to now [38,39,40,41,42,43,44,45,46].

**Figure 2:**
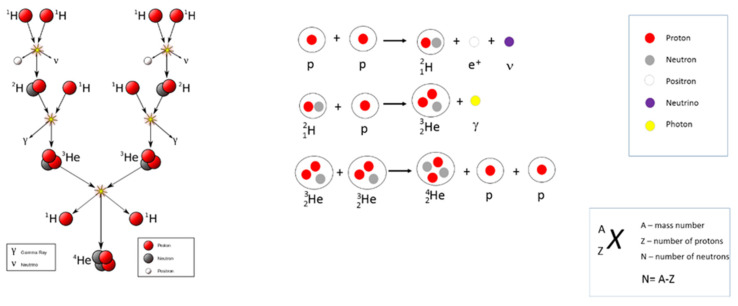
Fusion reaction scheme for the transformation of hydrogen into helium.

**Figure 3:**
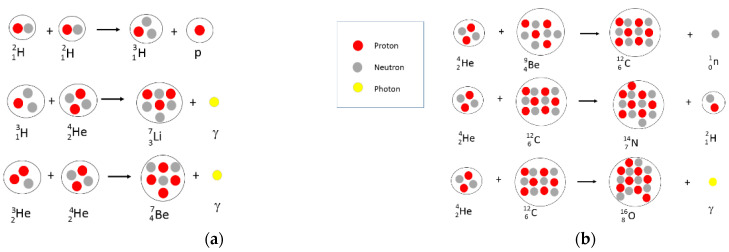
Hypothetic nucleosynthesis scheme for Li and Be production (**a**) and C, N, O possible nucleosynthesis (**b**).

In this hypothesis, the formation of the first periodic table elements after hydrogen such as helium (Z = 2), lithium (Z = 3), Be (Z= 4), C (Z = 6), N (Z = 7) etc., pass through successive fusion reactions as they are depicted in Figure 2 and Figure 3. This fusion scheme corresponds to the fusion nuclear reactions taking place in the Sun.

The nucleosynthesis of lithium, beryllium, and boron was, for a long time, difficult to explain due to the instability of these elements and their fragility at high temperature; this instability also being the cause of their very weak abundance in the “cosmic curve” of elements [47].

**Figure 4:**
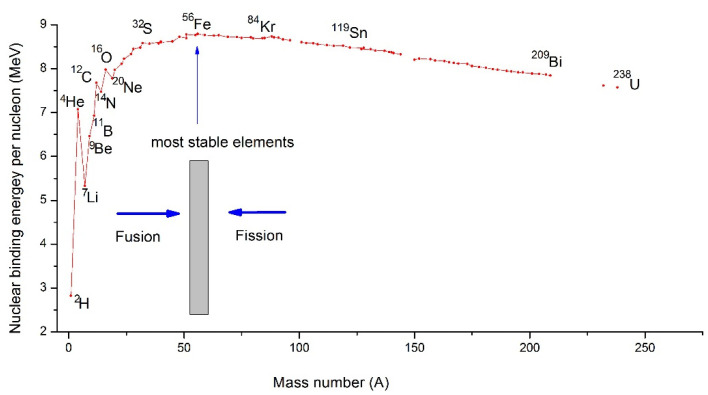
Nuclear binding energy per nucleon for each element in function of their mass number (A).

By analyzing the nuclear binding energy per nucleon (Figure 4) as a function of the atomic mass number of elements (A), it could be remarked that this energy presents a maximum for iron (Z = 26, A = 56). Considering that the most stable elements are those for which the binding energy per nucleon is highest, we can conclude that the lighter the elements, the higher the tendency to turn into heavy elements through fusion reactions, and elements with higher atomic mass tend to turn into lower atomic mass elements through fission reactions. Hence, this observation can also explain in a simple way the life cycle of stars and the abundance and scarcity of elements in the Universe.

**Figure 5:**
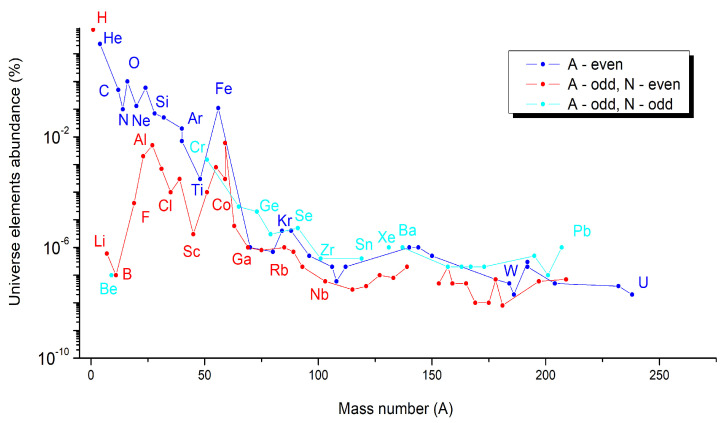
Universe-abundant elements as a function of their mass number (A) [49].

**Figure 6:**
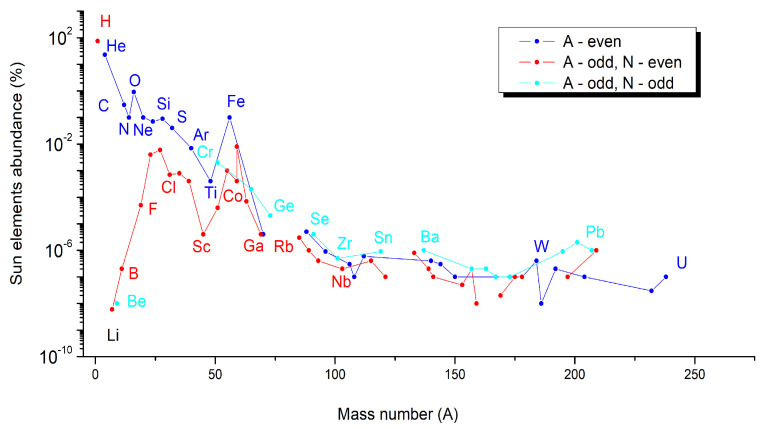
Sun-abundant elements as a function of their mass number (A) [49].

**Figure 7:**
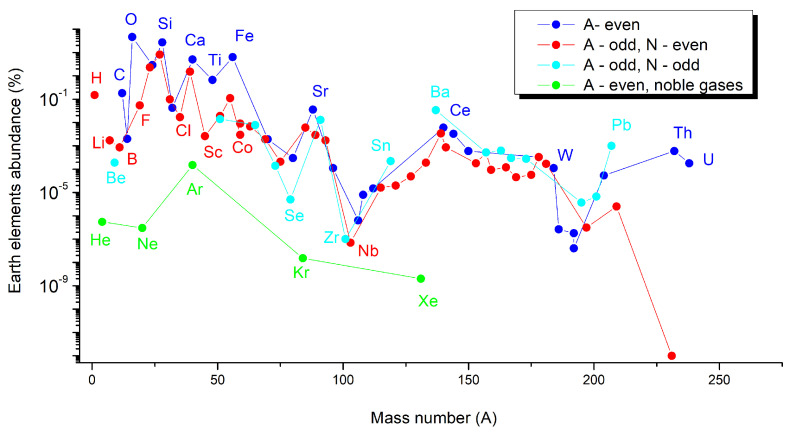
Earth-abundant elements as a function of their mass number (A) [49].

Based on astronomical, geological, and theoretical data [48,49,50,51,52,53], the abundance of elements at the Universe, Sun, or Earth scale was estimated for each element of the periodic table. Based on these data, we plot the graphs as a function of the even and odd A numbers of elements, but also by distinguishing for elements with odd A numbers, their odd or even number of neutrons (N). These graphs are given in the following:

(a) Figure 5 for Universe-abundant elements,
(b) Figure 6 for Sun-abundant elements, and
(c) Figure 7 for Earth-abundant elements.

A series of similarities are shown in these three graphs. In all cases, the elements having an even A number are more abundant (blue points) than the elements having an odd A number but an even number of neutrons (red points). This rule is respected including the noble gases in the case of the abundance of elements at the Universe and Sun scale (Figure 4 and Figure 5), but not for Earth, for which in the case of noble gases, even if they have an even A number, they are less abundant than all the other elements (Figure 6).

**Figure 8:**
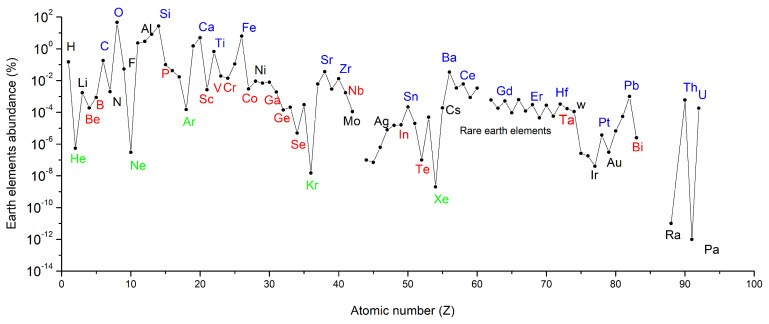
Earth-abundant elements as a function of their atomic number (Z) [49].

The abundance of elements on Earth as a function of their atomic number is given in Figure 8. Noble gases are represented in green and the other scarce elements are represented in red. It is not surprising to see that the scarce elements Be, B, P, Sc, V, Cr, Co, Ga, Ge, Se, Nb, In, and Te are rare earth elements; Ta and Bi are the same as the elements that were represented in Figure 1, presenting a supply and an economical risk. Many of the above-mentioned elements are also those that are continuously monitored by EU, and they are included in all the CRMs lists released since 2011.

These graphs in connection with the intrinsic properties of these elements at the nuclear scale (number of protons and number of neutrons) lead us to the idea that the properties of these elements may be very unique, and hence, these elements might be difficult to be replaced.

From this observation, the obvious conclusion is that although the substitution of CRMs represents an obvious solution (not at all easy to achieve), a more realistic option relates to a combination with rational use, enhanced recycling, sustainable mining, and reinvented products and processes.

## 3. From Solar Nebula to Planet Earth

Forming the chemical elements of our Universe was the first step. These clouds of gas and particles (solar nebula) accumulated and formed the proto-Earth about 4.54 Ga ago not showing significant order of the accumulated material, although the fractionation of refractory and volatile elements started [54]. Since then, the Earth passed several developing stages. Meteoroid impacts, radioactive decay, and further planetary compression led to enormous temperature melting the vast majority of the material. The melting point of Fe and Ni must have been reached to allow the metal–silicate fractionation that formed the Fe-Ni core [55], while the Earth’s rotation speed was much higher than it is today.

This early Earth was predominantly of mafic composition [56]. With time, the Earth further cooled down, forming a solid rigid crust that floats on a viscoelastic mantle, since crystal–liquid fractionation is considered as being a dominating fractionation process [57]. What is known as the giant impact model (GI) [58] has been simulating the collision of the proto-Earth with another Mars-sized [59] body or Mercury-sized [60] about 4.5 Ga ago. The GI model suggests that parts of the proto-Earth have been ejected and mingled with the impacting body while rotating around the Earth, forming discs and finally the Moon that helps to slows the rotation down, changed the inclination of the Earth’s axis, and keeps the Earth on a rather stable curve around the Sun and at today’s suitable distance and conditions. Those processes together with the ongoing plate tectonics caused by mantle convection leads to further separation of the chemical elements due to their physical properties and characteristics.

The Earth is still a dynamic planet that reworks its crust by different geological processes with variation in the rates of crustal reworking [61] and shifting from a highly mafic to a felsic bulk composition [62] through time.

Understanding the processes that formed the Earth of today and the behavior of the different elements is essential to discover unknown mineral deposits and for the processing of mined and waste material. The geological processes in combination with parameters such as pressure, temperature, time, fluid flow, and fugacity’s trigger the enrichment processes that might lead to significant enrichment that is technically and economically feasible. Analyses of natural rocks, experimental studies, and calculations are compiled and provide reference for bulk compositions, of which the bulk composition of the upper continual crust [63] is of upmost interest in the given context. In his research, Lehmann (2020) provides examples of the fractionation pattern during Earth’s history of which some are CRMs [57], indicating that exceptional processes needed to get significant anomalies. Geologists are looking for those outstanding features that might provide mineable future resources. The principle settings and European regions that host ore bodies enriched in siderophile (e.g., Fe, Ni, Pd) or chalcophile (e.g., Cu, In, Zn) elements have been described in projects such as FOREGS [64,65], also indicating the regions potential enriched in lithophile elements (e.g., Li, REE).

## 4. Principles on the Availability from EU Sources

The roughly 10.5 million km^2^ of Europe’s landmass is very diverse in terms of geology [66]. Regarding the remaining fragments of former continents, the different stages of reworked crust provide huge varieties in petrology of a differentiated crust that is composed of very unevenly distributed chemical elements [67]. These heterogeneities in the upper continental crustal composition range from about 3.8 to 3.9 Ga [68] and include old rocks of Greenland’s Greenstone Belt as remains of the North Atlantic Craton [69,70] and the correlated Lewisian complex in northwest Scotland [71], the Greenstone Belt and Tonalite–Trondhjemite–Granodiorites (TTG) fragments that remained in the Baltic Shield (part of Fennoscandia) and Ukraine Shield (part of Sarmatia) of the East European Craton (EEC) [72], and the regions of active volcanism. Tectonic settings play a key role when oceanic crust is merged with continental crust, in particular when oceans close and might expose volcanogenic massive sulfide (VMS) deposits [73]. The different setting contain a huge variety of deposit types close to Europe’s surface that have often been mined for centuries; among them are world-class producers of some economically important elements or even critical raw materials, including Pt, Sc, and Cr [57,74,75,76]. Yet, many minor-metal concentrations (by-products) were mostly not beneficiated at that time also due to inefficient technology and a lack of demand. The complex tectonic settings of the Balkan region and Greece add to the technological challenges for mining the ophiolite-related deposits.

From a geological perspective, Europe has an elevated raw materials potential even for many of the CRMs. For 13 out of 30 raw materials on the current EU list, part of the EU supply is provided by European countries (primary and secondary sources), among those Sr, for which Spain is one of the world-class producers. Yet, the risk for a disruptive value chain is even higher when the bottleneck in the value chain is already placed at the very beginning at the mining stage, as it is the case for Be, B, Co, Ta, and Sb listed also in Table 1 [4].

With the increasing attention to ensure robust raw material supply chains, more European deposits have been studied for their CRM supply potential, from Finland [77] to Greece [78,79], in central Europe [80,81], and from Poland [82] to Portugal [83,84]. With the right technology, the political and social agreement deposits can become mining sites, if economically feasible. A promising example that there might be more to mine is lithium, where the discovery of a new CRM-forming mineral (LiNaSiB_3_O_7_(OH) Jadarite) within a world-class deposit might be the stimulus to develop other deposits further [85].

Since geology does not follow or stop at political boarders, there is a need for transnational efforts to identify this potential. The EU has built up a couple of actions to strengthen the raw materials value chain for Europe, including basic research and technological developments, trade agreements, and the promotion of investments and financial schemes.

Among these actions are the activities of the Regional and National Geological Survey Organizations (GSOs) of the European States. General information on geotectonic and geochemical background facts on Europe are compiled. Principles for interactive GIS (geographic information system) tools and 3D/4D models of deposits and mineralized belts are developed by projects such as FOREGS, ProMINE, and Minerals4EU [65,86] as a one-off. However, the periodical update of Europe’s CRM list and other developments including land-use issues and the mining status in Europe calls for continues updating of validated, comparable, and timely updated information and the related maps provided through a publicly accessible and coordinated database. GeoERA Raw Materials takes advantage of the optimized network established by the GSOs, Europe’s long tradition in mining and quarrying, and new exploration methods, models, and data to unlock domestic resource potential. It comprises four projects ranging from dimension stone (EuroLithos) to seabed (MINDeSEA) and land-based (FRAME) minerals supported by a data management project (Mintell4EU). Based on the respective national databases on sites and commodities, GeoERA Raw Materials compiles and further unifies the geoscientific knowledge of Europe. Currently, 30 data providers from 29 European countries add to the established harvesting routine that collects, validates, and stores data in a central database on Europe’s resource potential, and more are about to join in 2021. The common and harmonized Minerals Inventory of known mineral resources, mining sites, and their status are visualized and publicly accessible.

**Figure 9:**
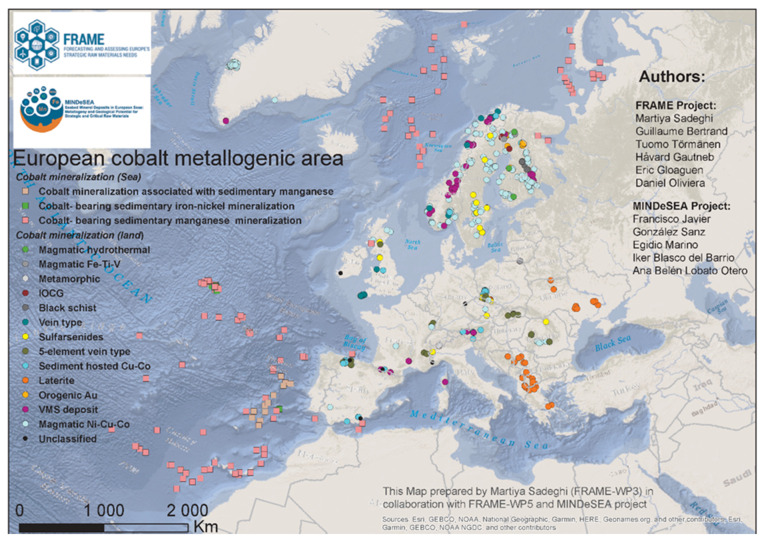
European cobalt metallogenic areas classified by style of mineralization, status October 2020, source: GeoERA scientific projects FRAME and MINDeSEA. GeoERA: Establishing the European Geological Surveys Research Area to deliver a Geological Service for Europe, FRAME: Forecasting And Assessing Europe’s Strategic Raw Materials Needs, MINDeSEA: Seabed Mineral Deposits in European Seas: Metallogeny and Geological Potential for Strategic and Critical Raw Materials. Reprinted from [13] Copyright From FRAME Project and MINSeSEA Project.

The identification and mapping of principal metallogenic areas that define models for different types of mineralization use modern and newly developed methods and technologies. Critical mineral potential mapping and quantitative mineral assessments on land and on the European seabed include the revisiting of residuals in EU historical mining sites. Based on a compilation of 509 Co-bearing deposits and occurrences identified in 25 European countries, a wide distribution for Co across Europe can be assumed that might be a viable future source [87]. The first Pan-European Map of Submarine Energy Critical Elements was compiled in 2018 [88] and has been improved periodically since 2020 [89]. Due to the societal needs, the first attempt put focus on the CRMs required for the energy transition (Figure 9). Together with the assessment of the European seabed, new potential sources of supply are identified. As part of the European Marine Observatory and Data Network (EMODnet), MINDeSEA adds to the comprehensive information of Europe’s maritime issues [90].

These new data and information merged with information on resource statistics (Minerals Yearbook), on demands, and others allow for strategic investments to develop Europe’s domestic resources that abide to the high ethical, social, and ecological standards that are further recurring recommendations of the European Commission.

The scientific research in these projects solidifies and innovates on existing premises, models, and strategies for mineral deposit and CRM exploration and establishes the first stepping stones to secure a reliable and responsible sourcing of mineral raw materials from domestic sources as viable future sources.

## 5. Conclusions: Which Solutions for Critical Raw Materials under Extreme Conditions?

In March 2020, the European Commission proposed to the European Parliament “A New Industrial Strategy for Europe” [91] to strengthen Europe’s open strategic autonomy by warning that with the transition of the European industry toward climate neutrality, current dependence on fossil fuels could be replaced by a dependence on raw materials. The communication says that the EU’s open strategic autonomy in these sectors will need to continue to be anchored in diversified and undistorted access to global commodity markets, but at the same time, it asserts that in order to reduce external dependencies and environmental pressures, it is necessary to address the underlying problem of rapidly increasing demand for global resources by reducing and reusing materials before recycling them.

Since the relevance of CRMs for industrial ecosystems is specific, and within the same industrial sector, their relevance in relation to the specific application is specific as well, if the aim is substitution, reduction, and recycling, it will be necessary to focus on a few specific raw materials.

This strategic assumption was the basis of the researcher’s consortium joined around the COST Action CA 15,102 CRM-EXTREME “Solutions for Critical Raw Materials Under Extreme Conditions”.

Practically, the researchers involved in the network were coordinated into four working groups that took on the issue with different approaches: (a) the first group studied why a particular element is so fundamental for the performances of a material; (b) the second group, on the basis of the knowledge of the first one, designed alternative materials; once they found an alternative material, (c) the third group developed the industrial process, and finally, (d) the fourth group dealt with the environmental and economic sustainability, including recycling issues, with a circular economy approach. This project strategy was demonstrated to be successful. This Special Issue collects the final project dissemination articles, and the advancement of the state of the art and solutions achieved by the partners of the CRM-ETREME Network can directly be appreciated.

## Figures and Tables

**Table 1:** A selection of the most economically important raw materials, mostly CRMs, listed in order of ascending atomic number (Z).

Atomic Number	Element	Main Production Country	Applications
Z = 3	Li	Chile, Australia, China, Argentina	Batteries
Z = 4	Be	United States, China, Kazakhstan	Foils used as radiation windows for X-ray detectors due to the low absorption of X-ray radiation High-speed aircrafts, satellites, and telescopes due to its stiffness and light weight Alloys with high electrical conductivity, high strength and hardness, corrosion, and fatigue resistant Mirrors Radio communications, nuclear applications, acoustic, electronics
Z = 5	B	Turkey, United States	Glass fibers, borosilicate glass with good resistance to thermal shocks Ceramics High-hardness and abrasive compounds Steels for nuclear industry Semiconductors Magnets Pharmaceutical applications
Z = 15	P	China, Morocco, US, Russia	Metallurgy, electronics (light-emitting diodes)
Z = 21	Sc	Ukraine, China, Russia	Aluminum alloys Mirrors for aerospace applications, lamps
Z = 22	Ti	China, Japan, Russia	Engines, alloys, paints, medical applications, aerospace industry, etc.
Z = 23	V	China, South Africa, Russia	Electrically conductive and thermal insulating metal, harder than most metals, with good resistance to corrosion, stable against strong acids; hence, it has a lot of application in industry Alloys as steel additive Catalysts
Z = 24	Cr ^1^	South Africa, Kazakhstan, India, Russia, Turkey	Metallurgy, steel alloys due to excellent high temperature properties, high hardness and corrosion resistance Pigments
Z = 27	Co	Congo, Russia, Australia, Canada, Rwanda	Alloys corrosion resistant for gas turbines and aircraft jet engines Magnets Batteries (LiCoO_2_) Catalysts Pigments and coloring (blue)
Z = 31	Ga	China, Japan, South Korea, Russia, Ukraine	Semiconductors Alloys
Z = 32	Ge	China, Russia, United States	Optical applications Electronics
Z = 34	Se ^1^	Germany, Japan, Belgium, Russia	Glass production Alloys Batteries, solar cells
Z = 38	Sr	China, Spain, Mexico	Radiopharmaceutical, cathode ray tubes
Z = 41	Nb	Brazil, Canada	Steel production Alloys and superalloys Superconducting magnets Electroceramics
Z = 49	In	China, South Korea, Japan, Canada	Semiconductors
Z = 51	Sb	China, Russia, Tajikistan, Bolivia	Flame retardants Alloys Semiconductors Catalyst
Z = 52	Te ^1^	Z = 52	Metallurgy Semiconductors, solar cells
Z = 73	Ta	Rwanda, Congo, Brazil	Electronics Alloys
Z = 74	W	China, Vietnam, Russia	Electrodes, heating elements, super-alloys, wear-resistant coatings, chemical applications, electronics etc.
Z = 83	Bi	China, Vietnam, Mexico	Pigments Alloys
Z = 57 to Z = 71	Rare Earth Elements	China	Catalyst Metallurgy Glass Ceramics

^1^ Not in EU CRMs list.

## Data Availability

The data presented in this study are available on request from the authors.
